# Osmotic Nephropathy Induced by L-Proline Stabilized Sucrose-free Intravenous Immunoglobulins: A Case Report

**DOI:** 10.1016/j.xkme.2025.101172

**Published:** 2025-11-04

**Authors:** Antonio Ulpiano Trillig, Samuel Rotman, Patricia Mehier, Alain Rossier, Gérard Vogel

**Affiliations:** 1 Unit of Nephrology, Riviera-Chablais Hospital, Rennaz, Switzerland; 2 Service of Clinical Pathology, Lausanne University Hospital and University of Lausanne, Lausanne, Switzerland; 3 Service of Nephrology and Hypertension, Lausanne University Hospital, Lausanne, Switzerland

**Keywords:** Osmotic nephropathy, proline tubular reabsorption, lysosomal accumulation, sucrose-free intravenous immunoglobulins, acute kidney injury, tubular cell vacuolization, pinocytosis, case report

## Abstract

Acute kidney injury following sucrose-free intravenous immunoglobulins (IVIG) is rare. We report the case of a 67-year-old male who developed a sudden anuric acute kidney injury at day 4 of L-proline stabilized sucrose-free IVIG for a Guillain-Barré Syndrome. The IVIG treatment was halted. The patient did not require renal replacement therapy after an adequate response to diuretics. Amoxicillin was the sole other potential nephrotoxic. The kidney biopsy showed typical features of osmotic nephropathy (ON). Although a certain degree of kidney hypoxia due to dysautonomia and variations of blood pressure might have occurred, histological findings were not compatible with an ischemic acute tubular necrosis. There was no glomerular and vascular involvement. Immunofluorescence of tubular cells cytoplasm was negative, ruling out antibody deposition. The patient had a complete renal recovery after 2 weeks. We hypothesize that proline itself acted as a reabsorbed toxic solute and accumulated in the lysosomes, leading to ON. In this case report we discuss the proline proximal tubular transport, involving pinocytosis in case of high concentration in the filtrate, and potential mechanisms involved in the development of ON.

Acute kidney injury (AKI) following administration of IVIG is well described with the use of sucrose containing IVIG.^1^ It is thought to be triggered by the proximal tubule reabsorption of a large amount of sucrose, notably by pinocytosis, with pinocytic vacuoles fusing with lysosomes.^1^^,^^2^ The accumulation of lysosomes in tubular cells, favored in the setting of altered lysosomal digestion such as a hypoxic state, leads to cellular dysfunction and swelling, lysosomal rupture, and ultimately AKI.^1^, ^2^, ^3^ A certain degree of tubular obstruction due to swelling of the tubular epithelium is possible.^2^ On electronic microscopy, a typical feature of this condition is lysosomal vacuoles containing amorphous debris.^2^^,^^4^ This process is termed osmotic nephropathy in the literature, although no osmotic effect is involved.^1^^,^^5^ It is described with the infusion of different agents such as mannitol, sorbitol, hydroxyethyl starch, contrast medium, and more recently with SGLT2 inhibitors, possibly involving glucose reabsorption in S3 segment of proximal tubule by pinocytosis.^1^^,^^2^^,^^4^^,^^5^

L-proline stabilized IVIG are very rarely associated with AKI. After administration of such IVIG, AKI is only described in the setting of hemoglobinuria and hemolytic anemia,^6^^,^^7^ but to our knowledge no case of osmotic nephropathy (ON) was previously reported.

We describe the case of an anuric AKI with biopsy-proven ON following L-proline stabilized sucrose-free IVIG perfusion.

### Case Report

A 67-year-old male with a medical history of treated arterial hypertension was admitted to the intensive care unit for an acute inflammatory demyelinating polyneuropathy following a SARS-CoV-2 infection. The infection began 17 days before his admission, with mild upper respiratory symptoms and a dry cough, already resolved on admission. There was no indication for remdesivir nor any SARS-CoV-2 infection specific treatment.

He was tetraparetic and presented wide variations of blood pressure due to dysautonomia, requiring intermittent use of intravenous nicardipine. He was treated with sucrose-free IVIGs for 4 days (Privigen, 20 g/day, infusion over 2 hours). The serum creatinine was stable between 59 and 67 μmol/L (0.67 to 0.76 mg/dL) from admission. On day 4 he developed a sudden anuric AKI. Serum creatinine level rose to 151 μmol/L (1.72 mg/dL) at day 5, and to 316 μmol/L (3.59 mg/dL) 12 hours later on the same day.

An indwelling bladder catheter was in place, and abdominal ultrasound ruled out obstructive nephropathy. Kidneys were normal in size and corticomedullar differentiation. Doppler analysis showed normal bilateral renal artery blood flow.

Intravenous furosemide was started, with a rapid rise of diuresis. The first interpretable urinalysis showed a proteinuria of 0.09 g/L, with an albuminuria of 31 mg/L, isomorphic hematuria and leukocyturia were present on urine microscopy, but no casts or crystals were visible.

Blood analysis showed a moderate thrombopenia but no hemolysis, with normal haptoglobin and lactate dehydrogenase levels, and no schistocytes in the blood smear analysis, ruling out a systemic thrombocytopenic microangiopathy. The C3 and C4 levels were in the normal range. Antineutrophil cytoplasmic antibodies anti-myeloperoxidase and anti-proteinase 3, antiglomerular basal membrane antibodies, and rheumatoid factor were negative, and antinuclear factor titer was < 160 1/DIL, with anti-native DNA antibody at borderline level (29.8 mUI/mL), which was considered a nonspecific finding. Cytomegalovirus serology realized at admission before IVIG treatment was positive for IgM and negative for IgG, but the blood polymerase chain reaction showed no detectable viremia.

A kidney biopsy was performed 8 days after the first dose of IVIG. Optical microscopy showed a massive vacuolization and swelling of proximal tubular cells and basal displacement of the nuclei compatible with an ON (Fig 1A and B). Proximal tubular cell brush borders were mostly well preserved, and no epithelium desquamation was apparent, although some mild quantity of vacuolized tubular cells with cytoplasm debris could be observed within luminal tubes. There was no glomerular or vascular involvement and no significant tubulo-interstitial fibrosis. Immunofluorescence of tubular cells cytoplasm was negative after preparation with pronase, ruling out antibody deposition. Electronic microscopy focused on tubular cells showed numerous intracytoplasmic lysosomal structures appearing as vacuoles (Fig 1C and D).

**Figure 1:**
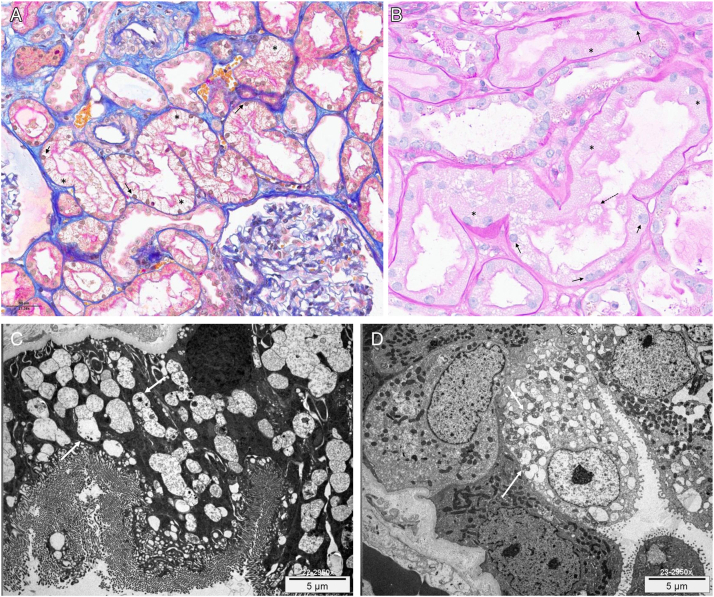
Osmotic nephropathy after infusion of L-proline stabilized IVIG. (A, Masson’s trichrome) and (B, PAS) Massive vacuolization of proximal tubular cells with foamy cytoplasm (∗), basal displacement of the nuclei (black arrow), preserved brush borders. Vacuolized tubular cells with cytoplasm debris within luminal tubes (dotted arrow). (C and D) Electronic microscopy showing numerous lysosomal structures appearing as vacuoles (white arrow).

Apart from IVIG, the only potential nephrotoxic was intravenous amoxicillin (2 g, 6 times/day) given to cover *Listeria Monocytogenes* meningitis while the PCR on cerebrospinal fluid was still pending at the time of AKI. As urinalysis did not show crystals and the biopsy did not show interstitial infiltrate, we did not consider amoxicillin as a likely culprit for the AKI. The IVIGs treatment was suspended.

At day 4 of AKI, the serum creatinine level rose up to 711 μmol/L (8.08 mg/dL), but without life-threatening metabolic acidosis, hyperkaliemia, or hypervolemia. There was therefore no need for renal replacement therapy. At day 7 of AKI, serum creatinine was 333 μmol/l (3.784 mg/dL) and at day 13 renal function had fully recovered with a serum creatinine of 88 μmol/L (1 mg/dL). The patient was then transferred to the neurological rehabilitation unit, where the neurological evolution was favorable.

## Discussion

AKI following L-proline stabilized IVIG was described in several case reports, following the development of hemolytic anemia with pigment-mediated kidney injury.^6^^,^^7^

In the case of our patient, a kidney biopsy showed a massive vacuolization of tubular cells, which appeared swollen and narrowed the lumen. There was a generalized clear cell appearance on optical microscopy, with a relatively low level of cell desquamation, mostly well-preserved brush borders, and basal displacement of the nuclei caused by the distended lysosomes (Fig 1A and B). Electronic microscopy showed numerous lysosomal structures (Fig 1C and D). These histological features are typical of ON.^1^^,^^3^^,^^5^^,^^8^ In ischemic acute tubular necrosis (ATN), the tubular epithelium is flattened, the lumen appears enlarged, and there is generalized loss of brush borders and epithelium desquamation.^9^ Even though cellular vacuolization can be present, it is typically not the dominant feature, and it is accompanied by other characteristics of ATN. Given the sudden anuria and severity of AKI in our patient, if ATN was responsible, we would expect a massive desquamation of the tubular epithelia, nuclear dropouts, and loss of brush borders, which were not present. Altogether these elements make the diagnosis of ON much more likely.

The pathophysiology of this injury thus appears to involve the massive tubular reabsorption of a product contained in Privigen and its accumulation in lysosomes, thus leading to tubular cell dysfunction and tubular obstruction by the swollen cells.

The hypothesis that the IgG themselves could be involved is unlikely because of the limited amount of intact immunoglobulins filtered through the glomerular basal membrane and podocyte slit diaphragm. The amount and type of proteinuria was not suggestive of glomerular injury, and the biopsy did not show any glomerular lesions; hence it is very unlikely that a sufficient amount of intact immunoglobulins could have been filtered and reached the proximal tubule leading to ON. In case of light chain nephropathy, the filtered light chains are massively reabsorbed by endocytosis in the proximal tubule as being ligand of megalin and cubilin.^10^ They form both intravacuolar and cytosolic crystals, often visible in optical microscopy and more accurately with electronic microscopy.^11^ The crystals are toxic for the proximal tubule cells. Light chains can also accumulate in lysosomes without crystal formation and trigger an inflammation via the production of reactive oxygen species and activation of JAK2/STAT1 pathway.^12^ In both cases, immunofluorescence after preparation with pronase highlights the light chains both in the cytosol and in the vacuoles. Cytosolic immunofluorescence staining was negative in the case of our patient, and electron microscopy did not show any crystal formation, ruling out the participation of immunoglobulins—intact or light chains—in the massive vacuolization of the proximal tubular cells.

We hypothesize that proline itself acted as a reabsorbed toxic solute and accumulated in the lysosomes, causing this massive vacuolization, similarly to sucrose, glucose, maltose, or sorbitol, for which the phenomenon is well described.^1^

**Figure 2:**
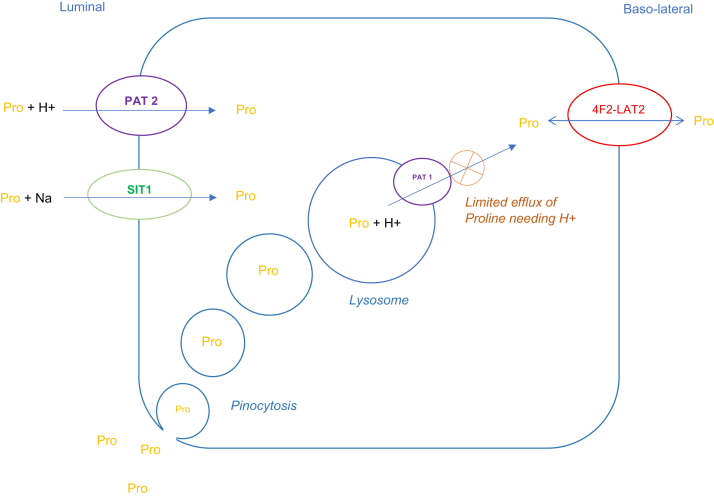
Proximal tubule transport of proline. Proline is freely filtered and fully reabsorbed luminally by the symporters SIT1 (SLC6A20, Na and Cl co-transport) and PAT2 (SLC36A2, H+-dependent transport). Basolaterally it is transported out (effluxed) mostly via the exchanger 4F2-LAT2 (SLC3A2-SLC7A8) that cooperates with uniporters. L-proline can efflux from lysosomes, probably mainly via PAT1 (SLC36A1), which is a H+-dependent symporter. If the transporters SIT1 and PAT2 are saturated, reabsorption of proline happens through pinocytosis and could accumulate in lysosomes due to the limited H+-dependent efflux through PAT1.

The usual concentration of L-proline in the filtrate is 100-300 μmol/l, the same as in the plasma, as it is freely filtered and reabsorbed luminally by SIT1 (SLC6A20, Na and Cl co-transport) and PAT2 (SLC36A2, a H+-dependent transport) symporters, a transport that is easily saturated^13^ It is transported out (effluxed) mostly via the basolateral exchanger 4F2-LAT2 (SLC3A2-SLC7A8) that cooperates with uniporters^13^ (Fig 2).

Higher proline plasma concentrations are encountered in hyperprolinemias, which are genetic inborn errors of the metabolism. They result from a deficiency in PRODH and P5 enzymes in type I and type II hyperprolinemias, and plasma proline concentrations range from 500 to 1,000 μmol/L and 1,500 to 3,000 μmol/L, respectively.^14^, ^15^, ^16^ These conditions are associated with congenital renal anomalies, deafness, and neurological disorders, but not with tubular injuries resulting from high proline concentration.^14^^,^^16^^,^^17^

Privigen contains a high concentration of proline (250 mmol/L). In the case of our patient, the dilution of the infused IVIG was standard at 20 g in 200 mL, and the rate of infusion was a standard 2 hour per infusion, with a progressively increased rate of infusion as per protocol. It exposed the patient to 50 mmol of proline per injection of 200 mL of Privigen, probably leading to higher plasma proline concentrations than in hyperprolinemia. The transient and repeated exposure to very high concentrations of proline might also have a different toxicity on tubular cells than stable mildly elevated levels. At this range of concentration in the filtrate, it is likely that the usual transport of proline is saturated and a further reabsorption happens through pinocytosis. The amount of solutes that enters the proximal tubular cells by pinocytosis depends on the quantity, rate, and duration of exposition.^1^

The kidney biopsy did not show any significant tubulo-interstitial fibrosis or other structural damage that could explain impaired tubular function, but the wide variations in blood pressure probably contributed to some degree to kidney hypoxia, a known critical factor limiting lysosomal digestion^1^ and proximal tubular cells metabolism.^12^ L-proline can normally efflux from lysosomes, probably mainly via PAT1 (Proton-assisted Amino acid Transporter 1, SLC36A1), a H^+^-dependent symporter^13^^,^^18^^,^^19^ (Fig 2). The capacity of L-proline efflux transport is probably limited, because of the necessary H^+^ transport into the lysosomes, and we can hypothesize that this step was impaired by kidney hypoxia, leading to the accumulation of proline in lysosomes and ultimately ON.

This case illustrates well the importance of administering IVIGs at a slow infusion rate, particularly in hemodynamically unstable patients with chronic kidney disease or exposed to other proximal tubule toxics. Clinicians must be aware that all types of IVIGs can cause ON.
